# Miocardite por COVID-19 Mimetizando Infarto Miocárdico com Supradesnivelamento de Segmento ST

**DOI:** 10.36660/abc.20210749

**Published:** 2022-07-13

**Authors:** Anthony Medina Conceição, César A. C. Pereira, Maria Júlia Rahal, Walther Yoshiharu Ishikawa, Carlos E. Rochitte

**Affiliations:** 1 Hospital das Clínicas Faculdade de Medicina Universidade de São Paulo São Paulo SP Brasil Instituto do Coração do Hospital das Clínicas da Faculdade de Medicina da Universidade de São Paulo, São Paulo, SP – Brasil

**Keywords:** Miocardite, Síndrome Coronariana Aguda, COVID-19, Imagem por Ressonância Magnética

## Introdução

O novo SARS-CoV-2, causador da COVID-19 ( *coronavírus disease 2019* ), provou-se bem mais que um vírus que acomete o sistema respiratório isoladamente. Na doença, ocorrem implicações sistêmicas diversas, incluindo manifestações cardiovasculares.^1 , 2^ Pacientes com doença cardiovascular prévia e que desenvolvem injúria miocárdica evoluem com piores desfechos,^2 , 3^ tais como síndrome coronariana aguda (SCA)^4 , 5^ e miocardite.^6 - 8^ Os quadros de miocardite são predominantemente assintomáticos, mas podem se apresentar com dor torácica anginosa, falência cardíaca aguda e arritmias.^9 - 12^

O diagnóstico clínico de miocardite sem utilização de avaliação complementar específica dificilmente é possível. Uma metanálise com 2866 pacientes diagnosticados com infarto miocárdico sem lesões obstrutivas (MINOCA) que realizaram ressonância magnética cardíaca (RMC) encontrou uma prevalência de miocardite de 34,5%.^11^ No cenário da COVID-19, um estudo na Alemanha detectou que 60% dos pacientes recentemente recuperados possuíam sinais de inflamação miocárdica na RMC.^13^

## Relato de caso

Paciente do sexo masculino, 43 anos, sem comorbidades, foi admitido em unidade de pronto socorro na atenção primária. Relatava episódios de dor anginosa típica há cinco dias, retroesternal, irradiada para membro superior esquerdo, desencadeada por esforços, com alívio ao repouso, duração de poucos minutos, com dispneia classe funcional II associada. No dia do atendimento, evoluiu durante esforço com dor de mesmo padrão com forte intensidade, incapacitante, sem melhora ao repouso. Foi admitido na unidade com cerca de uma hora do início da dor. Referiu quadro gripal iniciado dois dias antes do primeiro episódio de dor, com temperatura de 37,7°C. Sua esposa teve quadro gripal iniciado 10 dias antes, tendo diagnóstico confirmado de COVID-19.

Ao exame físico, apresentava-se lúcido, orientado, eupneico, com saturação periférica de oxigênio de 98% em ar ambiente, afebril, frequência cardíaca de 80 batimentos por minuto, pressão arterial de 120x90 mmHg. Sem alterações em ausculta cardíaca e pulmonar, ou no exame abdominal. Sem sinais de congestão.

**Figura 1:**
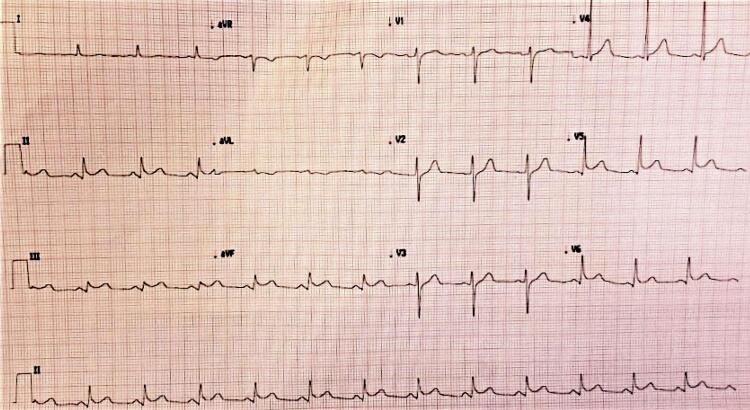
Eletrocardiograma do primeiro atendimento, mostrando supradesnivelamento de segmento-ST em paredes inferior e lateral.

Eletrocardiograma (ECG) de 12 derivações ( Figura 1 ), evidenciava ritmo sinusal, com supradesnivelamento de segmento ST de 2mm em parede inferior (D2,D3 e aVF) e antero-lateral (V4-V6). Recebeu dupla antiagregação com ácido acetilsalicílico (AAS) e clopidogrel e anticoagulação com enoxaparina. Foi realizada trombólise com alteplase na unidade, após duas horas do início da dor, com melhora parcial e manutenção do supra-ST.

Cerca de oito horas da trombólise, o paciente foi transferido para hospital terciário. Foi realizado cateterismo, com ausência de ateromatose ou trombos intracoronários e ventriculografia normal. Primeira troponina ultrassensível >25.000 ng/L (VR <58 ng/L) e CK-MB massa 96 ng/mL (VR <4,4 ng/mL). Radiografia de tórax evidenciava hipotransparência discreta em bases pulmonares. Diante da suspeita de COVID-19, foi realizado teste rápido para antígeno, com resultado negativo, além de RT-PCR para SARS-CoV-2 em swab orofaríngeo, negativo em duas testagens em dias diferentes.

**Figura 2:**
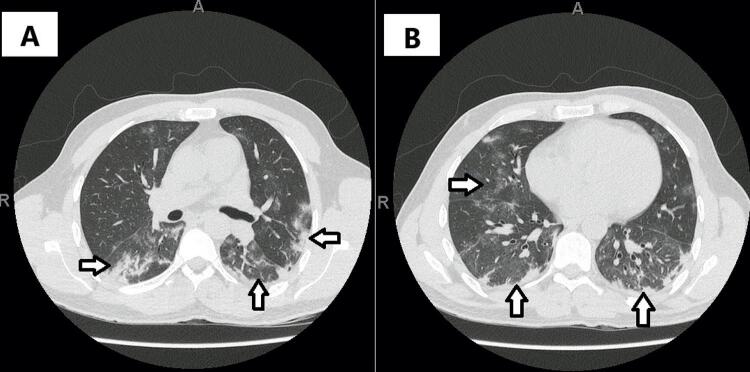
Imagem do tórax por tomografia computadorizada, evidenciando múltiplas opacidades em vidro fosco esparsas e bilaterais, predominantes nos campos pulmonares inferiores, compatíveis com acometimento por COVID-19. A) é possível notar o acometimento dos lobos inferiores direito e esquerdo, bem como da porção mais inferior do lobo superior esquerdo. B) destacam-se, além das áreas de vidro fosco, a ocorrência de pequenos focos confluentes consolidativos periféricos nos lobos inferiores.

O paciente foi submetido a ecocardiograma, que mostrou fração de ejeção de ventrículo esquerdo preservada (65%), sem alterações segmentares das paredes. Tomografia computadorizada de tórax ( Figura 2 ) evidenciou múltiplas opacidades focais e em vidro fosco esparsas e bilaterais, mais evidentes em bases pulmonares, compatíveis com pneumonia viral, inclusive COVID-19. A extensão do acometimento pulmonar foi estimada em 25-50%.

**Figura 3:**
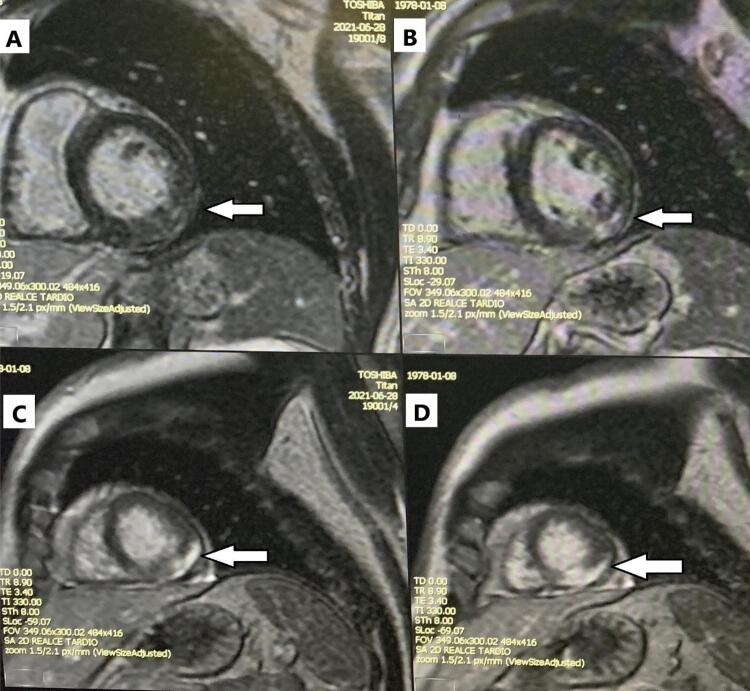
Ressonância magnética cardíaca evidenciando realce tardio pelo gadolínio de padrão não transmural, com predomínio mesoepicárdico e subepicárdico, compatível com miocardite. Realce acometendo o segmento basal (A) e médio (B) da parede ínfero-lateral; segmentos apicais das paredes inferior e lateral (C) e segmento apical da parede inferior (D). As regiões acometidas estão identificadas por setas brancas.

Considerando-se o quadro de SCA com supra-ST, e ausência de lesões coronárias ou de disfunção sistólica segmentar, no quarto dia de internação foi optado por realização de RMC ( Figura 3 ). Foram identificadas áreas de realce tardio miocárdico de padrão não isquêmico, em segmentos médio e basal da parede ínfero-lateral, e apicais das paredes lateral e inferior, com tênue edema miocárdico, sugerindo miocardite aguda. Não foi realizada avaliação quantitativa com mapeamento paramétrico T1 ou T2.

O paciente evoluiu sem complicações e recebeu alta no sexto dia de internação para seguimento ambulatorial. Neste momento, após revisão do laudo da RMC, foi suspensa a dupla antiagregação plaquetária, e optado por manutenção de atorvastatina. A Tabela 1 resume a evolução dos exames laboratoriais ao longo da internação. O ECG não apresentou evolução típica de infarto, evidenciado por alta manutenção do ritmo sinusal com supra-ST em V4-V6 e em D2, com alteração da repolarização em D3 e AVF. Sorologia para SARS-CoV-2 coletada no dia da alta foi positiva (874 unidades de anticorpo ligado/mL, VR ≥ 33,8/mL - padrão OMS). O paciente não havia recebido nenhuma vacina para COVID-19.

**Tabela 1:** Evolução temporal dos exames laboratoriais ao longo dos dias de internação

	Primeiro dia	Segundo dia	Quarto dia	Sexto dia	VR*
Troponina, ng/L	> 25000	10128	4565	2330	< 58
CK-MB, ng/mL		96	9,2	0,61	< 4,4
Creatinina, mg/dL		0,83	0,98	0,77	0,7 - 1,3
Ureia, mg/dL		25	41	25	15 - 39
Sódio, mmol/L	137	136	139	140	136 - 145
Potássio, mmol/L	3,5	4,0	4,0	4,6	3,5 - 5,0
Magnésio, mg/dL	2,5	2,3	2,1	2,1	1,8 - 2,4
Proteína-C reativa, mg/L		35,3	9,7	4,6	< 5
Hemoglobina, g/dL		12,3	10,3	11,3	13,5 - 17,5
Hematócrito, %		37	31	34	39 - 50
Leucócitos, U/mm³		10800	9360	10200	3500 - 10500
Plaquetas, U/mm³		818000	699000	612000	150000 - 450000
Colesterol total, mg/dL				205	< 190
HDL colesterol, mg/dL				23	> 40
LDL colesterol, mg/dL				104	< 130
Triglicérides, mg/dL				388	< 150
Hemoglobina glicada, %				5,0	< 5,7

*VR: valor de referência; HDL: lipoproteína de alta densidade; LDL: lipoproteína de baixa densidade

## Discussão

Diante da heterogeneidade de apresentação, o diagnóstico de miocardite continua sendo um desafio.^12^ O mesmo ocorre em pacientes com SCA e diagnóstico presumido de infarto sem alterações coronarianas que o justifiquem.^14^ Diversos estudos com RMC demonstram que a maioria desses pacientes na verdade possuem miocardite.^14 , 15^

No Reino Unido, 79 pacientes admitidos por SCA com elevação de troponinas sem lesão em angiografia foram submetidos à RMC. Desses pacientes, 81% foram diagnosticados com miocardite, com edema miocárdico em 58% e realce compatível em 92%.^15^ Em outro estudo inglês, 60 pacientes foram submetidos à ressonância até três meses após episódio de dor torácica, com troponinas elevadas e cateterismo sem lesões obstrutivas. Em 65% dos casos chegou-se a um diagnóstico, sendo 50% miocardite. Desses pacientes, 40% apresentaram elevação do segmento ST e 31% foram trombolisados.^14^ A melhora da dor com uso de trombolítico não é bem explicada, porém não implica em relação de causa-efeito. O paciente já havia apresentado quadro de dor torácica com resolução espontânea nos dias que precederam o pico da dor, além de não haver evolução temporal típica de infarto no ECG.

O padrão-ouro ainda aceito para o diagnóstico de miocardite é a biópsia endomiocárdica.^9 , 12 , 16^ No entanto, seu caráter invasivo, possibilidade de complicações, pouca disponibilidade e limitações diagnósticas desestimulam seu uso rotineiro, principalmente em casos não graves, tal qual o caso descrito. A RMC já está bem estabelecida como opção de avaliação não invasiva para tal finalidade.^9 , 12 , 14 - 16^ Este método combina segurança, avaliação anatômica, consistência interobservador e acurácia quantitativa, fornecendo compreensão diagnóstica de diversas entidades.^16^

A Sociedade Europeia de Cardiologia (ESC) sugere critérios clínicos e alterações de exames complementares não invasivos, como ECG, troponina, ecocardiograma e RMC, para diagnóstico de miocardite, tornando a biópsia endomiocárdica não necessariamente obrigatória.^12^ Para o uso da RMC no diagnóstico de miocardite, utilizam-se os critérios de Lake Louise, que envolvem: 1 - a avaliação da intensidade do sinal miocárdico em T2 compatível com edema; 2 - o realce miocárdico global precoce pós contraste de gadolínio em T1; e 3 – o realce miocárdico tardio pós contraste de gadolínio em T1.^12 , 16^ O padrão de lesão após insulto isquêmico é de caráter transmural, necessitando acometimento subendocárdico para tal caracterização. O padrão não-isquêmico varia de não-transmural, preferencialmente mesocárdico e subepicárdico, podendo ser multifocal, ou até mesmo transmural, o que dificulta a diferenciação neste último caso.^12 - 16^

A apresentação de COVID-19 com SCA foi documentada,^4 , 5^ e associada a um pior prognóstico. Em estudo brasileiro, a taxa de mortalidade hospitalar foi de 23,7%, no qual 12,5% dos 152 pacientes não possuíam lesões obstrutivas.^5^ Em pequeno estudo italiano, 40 % dos pacientes com SCA não possuíam doença coronária obstrutiva, com mortalidade de 40% em seguimento médio de duas semanas. Desses pacientes, 85% não possuíam sintomas respiratórios ou testes positivos para COVID-19 no momento do cateterismo, tendo SCA com supra de ST como primeira manifestação clínica do COVID-19.^4^

A injúria miocárdica é fortemente correlacionada com pior prognóstico da COVID-19, inclusive desfechos fatais.^1 - 3 , 17^ A incidência de miocardite pelo SARS-CoV-2 ainda é desconhecida, embora diversos casos estejam relatados.^1 , 6 , 7 , 13 , 17 , 18^

Trazemos relato de um paciente com COVID-19 que evolui com SCA com supra-ST, submetido à trombólise com cateterismo sem lesões obstrutivas, e sem alterações ecocardiográficas, finalmente diagnosticado com miocardite por RMC. As consequências a longo prazo também são desconhecidas, reforçando a necessidade de estudos de seguimento.^7 , 17^

O diagnóstico de miocardite não é óbvio diante de um quadro de dor torácica anginosa com alteração de ECG e elevação de troponina, sendo necessária a exclusão de doença coronária pelo cateterismo, para preencher os critérios atuais de definição de MINOCA.^11 , 19^ Uma vez não alcançado o diagnóstico, é recomendado que se prossiga com a investigação etiológica, de preferência com a realização de RMC.^19^ Não há consenso quanto ao momento ideal ou precocidade de realização do exame, no entanto, é factível sua realização tão logo seja obtida a estabilidade clínica do paciente. Este relato contempla diversas entidades clínicas envolvidas no desafio diagnóstico que é a miocardite, reforçando o papel essencial da RMC nessa apresentação clínica, de um paciente com COVID-19 sem doença coronariana prévia, que desenvolveu SCA com supra de ST.
