# Supplementary material for: Miocardite por COVID-19 Mimetizando Infarto Miocárdico com Supradesnivelamento de Segmento ST
Source: Arq Bras Cardiol. 2022 Jul 13;119(3):480–4. [Article in Portuguese] doi: 10.36660/abc.20210749 (PMC9438540; doi:10.36660/abc.20210749)
Supplement: Translation

# Translation: COVID-19 Myocarditis Mimicking ST-Segment Elevation Myocardial Infarction

**Authors:** Anthony Medina Conceição, César A. C. Pereira, Maria Júlia Rahal, Walther Yoshiharu Ishikawa, Carlos E. Rochitte

**Affiliations:** 1 Brasil Instituto do Coração do Hospital das Clínicas da Faculdade de Medicina da Universidade de São Paulo, São Paulo, SP – Brasil

**Keywords:** Myocarditis, Acute Coronary Syndrome, COVID-19, Magnetic Resonance Imaging

## Introduction

The new SARS-CoV-2, that causes the coronavirus disease 2019 (COVID-19) has proven to be a virus that affect not only the respiratory system, but to cause several systemic manifestations, including cardiovascular ones.^1 , 2^ Patients with previous cardiovascular disease who develop myocardial injury usually have worse outcomes,^2 , 3^ such as acute coronary syndrome (ACS)^4 , 5^ and myocarditis.^6 - 8^ Myocarditis is mostly asymptomatic but can manifest with angina, cardiac failure, and arrythmias.^9 - 12^

The clinical diagnosis of myocarditis without the aid of complementary exams is usually difficult to be made. A meta-analysis with 2,866 with myocardial infarction without obstructive coronary artery disease (MINOCA) who underwent cardiac magnetic resonance (CMR) showed a prevalence of myocarditis of 34.5%.^11^ In COVID-19, a study carried out in Germany reported that 60% of recently recovered patients had signs of myocardial inflammation at CMR.^13^

## Case report

Male patient, 43 years old, without comorbidities, admitted to a primary care emergency. The patient complained of typical angina in the form of retrosternal pain radiating to the left arm, for five days, triggered by exertion and relieved with rest, lasting a few minutes, associated with functional class II dyspnea. On admission day, the patient had strong, debilitating pain of the same pattern during exertion, with no improvement with resting. The pain had started about one hour before admission. The patient reported a flu-like illness two days before the first episode of pain, temperature of 37.7^o^C. His wife had a flu-like illness initiated ten days before and had received a diagnosis of COVID-19.

On physical examination, the patient was conscious, fully oriented, eupneic, with peripheral oxygen saturation of 98% on room air, afebrile, heart rate of 80 bpm, blood pressure of 120x90mmHg, normal cardiac and pulmonary auscultations, normal abdominal examination, and no signs of congestion.

Twelve-lead electrocardiogram (ECG) ( Figure 1 ) showed sinus rhythm with 2-mm inferior wall (D2, D3 and aVF) and anterolateral ST-segment elevation (V4-V6). The patient received dual antiplatelet therapy with acetylsalicylic acid (ASA) and clopidogrel, and enoxaparin for anticoagulation, and antithrombotic therapy with alteplase two hours after pain onset. The patient had partial improvement, but the ST-segment elevation was maintained.

**Figure 1:** Electrocardiogram on admission, showing inferior and lateral wall ST-segment elevation.

Approximately eight hours after thrombolysis, the patient was transferred to a tertiary hospital. The patient underwent catheterization, which revealed no coronary atheroma or thrombosis, and normal ventriculography. The first high-sensitivity troponin was >25,000 ng/L (VR <58 ng/L) and CK-MB mass of 96 ng/mL (VR <4.4 ng/mL). Chest X-ray revealed little opacity of lung bases. Due to suspected COVID-19, a rapid antigen test was performed, with a negative result, in addition to two RT-PCR tests for SARS-CoV-2 (oropharyngeal swab) on separate days, with negative results.

The patient underwent echocardiography, which showed preserved ejection fraction (65%), with no segmental wall motion abnormalities. Chest computed tomography ( Figure 2 ) revealed bilateral ground-glass opacities, predominantly in lung basis, compatible with viral pneumonia, including COVID-19. The extent of pulmonary involvement was estimated as 25-50%.

**Figure 2:** Chest computed tomography showing bilateral ground-glass opacities, predominantly in lung basis, compatible with COVID-19. Involvement of right and left lower lobes, and the lower portion of the right upper lobe (A); areas of ground-glass opacities and peripheral confluent consolidation in lower lobes (B).

Considering that the patient had ST-elevation ACS and absence of coronary lesions or segmental systolic dysfunction, on the fourth day of hospitalization, CMR was performed ( Figure 3 ). Non-ischemic delayed myocardial enhancement was detected, in the mid and basal segments of the lower lateral wall, and in the apical segment of the lateral and inferior walls, mild myocardial edema, suggestive of acute myocarditis. Quantitative analysis with parametric (T1 and T2) mapping was not performed.

**Figure 3:** Cardiac magnetic resonance showing late gadolinium enhancement of non-ischemic pattern, predominantly in mid-myocardium and subendocardium, compatible with myocarditis. Delayed myocardial enhancement in the basal (A) and mid (B) segments of the lower lateral, apical segment of the lateral and inferior walls (C), apical segment of the inferior wall (D). Areas affected are indicated by white arrows.

The patient had a good clinical course, without complications, and was discharged on the sixth day of hospitalization for outpatient follow-up. The CMR result was reviewed, and it was decided to discontinue dual antiplatelet therapy and to continue atorvastatin. ECG did not show the typical pattern of infarction, evidenced by the maintenance of sinus rhythm with ST elevation in V4-V6 and D2, and change in repolarization in D3 and AVF. The patient had a positive SARS-CoV-2 antibody test on day of discharge (874 units of bound antibodies/mL, VR ≥ 33.8/mL – WHO standards). The patient had not been vaccinated against COVID-19.

## Discussion

Because of its heterogenous presentation, the diagnosis of myocarditis remains a challenge.^12^ The same occurs in patients with ACS and a presumed diagnosis of infarction but with no coronary changes that explain it.^14^ Several studies with CMR have shown that most of these patients have in fact myocarditis.^14 , 15^

In the United Kingdom, 79 patients admitted for ACS with elevation of troponin levels and no injury at angiography were submitted to CMR. Of these patients, 81% were diagnosed with myocarditis, with myocardial edema in 58% and compatible enhancement in 92%.^15^ In another English study 60 patients were submitted to CMR within three months of the episode of chest pain, with increased troponin and no obstructive lesions at catheterization. A diagnosis was established in 65% of cases, and 50% of patients had myocarditis. Of these patients, 40% had elevation of ST segment and 31% received thrombolytic treatment.^14^ Although the improvement of pain with thrombolytic agents is not well explained, a cause-effect relationship is not implied. The patient had already experienced chest pain with spontaneous resolution for days before the worst pain episode, and no typical temporal pattern of infarction was seen on ECG.

Endomyocardial biopsy is still the gold standard for the diagnosis of myocarditis.^9 , 12 , 16^ Nevertheless, due to its invasive nature, potential complications, low availability and diagnostic limitations, the procedure is not performed routinely, especially in non-severe cases, as in this reported case. CMR has already been well established as a non-invasive alternative for this purpose.^9 , 12 , 14 - 16^ This method combines safety, anatomical assessment, consistency between observers, and quantitative accuracy, providing diagnostic information in many diseases.^16^

The European Society of Cardiology (ESC) suggests clinical criteria and reference results for non-invasive complementary tests (e.g., ECG, troponin, echocardiogram and CMR) for the diagnosis of myocarditis, making the endomyocardial biopsy not necessarily mandatory.^12^ The Lake Louise criteria are the diagnostic CMR imaging criteria for myocarditis and involve: 1- measurement of myocardial signal intensity in T2 compatible with edema; 2- early gadolinium enhancement in T1; and 3- late gadolinium enhancement in T1.^12 , 16^ The pattern of injury after an ischemic insult is characterized by transmural progression, including the subendocardium. The non-ischemic pattern varies from non-transmural, mainly mid-myocardial and subendocardial, multifocal, until transmural, which may make differentiation difficult.^12 - 16^

The presentation of COVID-19 with ACS has been documented,^4 , 5^ and associated with a poor prognosis. In a Brazilian study, hospital mortality rate was 23.7%; 12.5% of 152 patients did not have obstructive lesions.^5^ In a small Italian study, 40% of ACS patients did not have obstructive coronary disease, with a mortality of 40% in a mean follow-up period of two weeks. Of these patients, 85% did not have respiratory symptoms or positive test for COVID-19 at the time of catheterization, with ST elevation ACS the first clinical manifestation of COVID-19.^4^

**Table 1:** Temporal progression of laboratory test results during hospitalization

	First day	Second day	Fourth day	Sixth day	RV*
Troponin, ng/L	> 25000	10128	4565	2330	< 58
CK-MB, ng/mL		96	9.2	0.61	< 4.4
Creatinine, mg/dL		0.83	0.98	0.77	0.7 - 1.3
Urea, mg/dL		25	41	25	15 - 39
Sodium, mmol/L	137	136	139	140	136 - 145
Potassium, mmol/L	3.5	4.0	4.0	4.6	3.5 - 5.0
Magnesium, mg/dL	2.5	2.3	2.1	2.1	1.8 - 2.4
C-reactive protein, mg/L		35.3	9.7	4.6	< 5
Hemoglobin, g/dL		12.3	10.3	11.3	13.5 - 17.5
Hematocrit, %		37	31	34	39 - 50
Leukocytes, U/mm^3^		10800	9360	10200	3500 - 10500
Platelets, U/mm^3^		818000	699000	612000	150000 - 450000
Total cholesterol, mg/dL				205	< 190
HDL cholesterol, mg/dL				23	> 40
LDL cholesterol, mg/dL				104	< 130
Triglycerides, mg/dL				388	< 150
Glycated hemoglobin, %				5.0	< 5.7

*RV: reference value HDL: high-density lipoprotein; LDL: low-density lipoprotein.

Myocardial injury is strongly correlated with a worse prognosis of COVID-19, including fatal outcomes.^1 - 3 , 17^ The incidence of myocarditis caused by SARS-CoV-2 is still unknown, despite several cases reported.^1 , 6 , 7 , 13 , 17 , 18^

We report a case of a COVID-19 patient who developed with ST elevation ACS, underwent thrombolysis with catheterization, with no obstructive lesions and no echocardiographic changes, and a final diagnosis of myocarditis determined by CMR. The long-term consequences are also unknown, reinforcing the need for follow-up studies.^7 , 17^

The diagnosis of myocarditis is not obvious in the case of angina with electrocardiographic changes and elevation of troponin, requiring the exclusion of coronary disease by catheterization, to fulfill the current criteria of MINOCA.^11 , 19^ Once the diagnosis could not be established, it is recommended to continue with the etiologic investigation, preferably with CMR.^19^ There is no consensus on the best moment or how early CMR should be performed, but it is known that the test is feasible as soon as the patient is clinically stable. This report addresses several clinical conditions involved in the diagnostic challenge of myocarditis, reinforcing the role of CMR in this case, of a COVID-19 patient with no history of coronary disease, who developed ST elevation ACS.
